# Epidemiologic characteristics, clinical management, and public health implications of Coronavirus Disease 2019 (COVID-19) in pregnancy: A Systematic Review and Meta-analysis

**DOI:** 10.3126/nje.v11i4.41911

**Published:** 2021-12-31

**Authors:** Brijesh Sathian, Indrajit Banerjee, Ahammed A Mekkodathil, Edwin R. van Teijlingen, Ana Beatriz Pizarro, Mohammad Asim, Maraeh Angela Mancha, Russell Kabir, Padam Simkhada, Israel Júnior Borges do Nascimento, Hanadi Al Hamad

**Affiliations:** 1,7,11 Geriatrics and long term care Department, Rumailah Hospital, Hamad Medical Corporation, Doha, Qatar; 2 Sir Seewoosagur Ramgoolam Medical College, Mauritius; 3,6 Trauma Surgery, Hamad General Hospital, Doha, Qatar; 1,4,9 Bournemouth University, Bournemouth, England, United Kingdom; 5 Clinical Research Center, Fundación Valle del Lili. Cali, Colombia; 8 School of Allied Health, Faculty of Health, Education, Medicine, and Social Care, Anglia Ruskin University, Chelmsford, United Kingdom; 9 University of Huddersfield, United Kingdom; 10 University Hospital and School of Medicine, Federal University of Minas Gerais, Belo Horizonte, Minas Gerais, Brazil; 11 Medical Director, Rumailah Hospital (RH) & Qatar Rehabilitation Institute (QRI), Doha, Qatar

**Keywords:** Pandemic, Pregnancy, Childbirth, Complications, Clinical Management, Prevention

## Abstract

**Background:**

The novel Coronavirus Disease 2019 (COVID-19) outbreak, caused by the pathogenic severe acute respiratory syndrome-2 (SARS-CoV-2) virus, is exponentially spreading across the globe.

**Methods:**

The current systematic review was performed utilising the following electronic databases PubMed, MEDLINE and EMBASE. We searched for the keywords "COVID-19 AND "pregnancy" between January 1, 2020 until December 31, 2020.

**Results:**

Out of 4005 records which were identified, 36 original studies were included in this systematic review. Pooled prevalence of vertical transmission was 10%, 95% CI: 4-17%. Pooled prevalence of neonatal mortality was 7%, 95% CI: 0-21%.

**Conclusion:**

The contemporary evidence suggests that the incubation period of COVID-19 is 2-14 days, and this infection could be transmitted even from the infected asymptomatic individuals. It is found that the clinical presentation of pregnant women with COVID-19 infection is comparable with the infected non-pregnant females, and the frequent symptoms were fever, cough, myalgia, sore throat and malaise. Some cases have severe maternal morbidity and perinatal deaths secondary to COVID-19 infection. Under these circumstances, pregnant women should focus on maintaining personal hygiene, proper nutrition and extreme social distancing to reduce the risk of COVID-19. Therefore, systematic data reporting for evidence based clinical assessment, management and pregnancy outcomes is essential for preventing of COVID-19 infection among pregnant women.

## Introduction

The current pandemic of novel coronavirus disease 2019 (COVID-19) has accounted for more than 3 million infections with over 210,000 infection-related deaths worldwide [1]. It is considered a severe public health emergency due to a higher rate of infectivity and deadly nature affecting all ages and both genders. Particularly vulnerable populations including pregnant women, the elderly, adults with co-morbidities and paediatrics, are at increased risk of complications. Inadequate healthcare resources and workforce can further compromise the care of vulnerable individuals. The substantial morbidity associated with the disease and potential socioeconomic impact necessitates the implementation of drastic preventive measures across all continents, such as nationwide lockdowns and closure of borders. Notably, the current evidence suggests that COVID-19 infection has significant impacts on pregnant women and foetuses similar to the risk of complications associated with the 2009 pandemic of H1N1 influenza virus among pregnant women and the severe effects of Zika virus infections on foetal outcomes [2-5]. Concerning the maternal immune system during pregnancy, there is a cell-mediated immunity shift from T-helper 1 (Th1) towards the proliferation of T-helper 2 (Th2) cells which safeguard the foetus, but at the same time keeps the mother more vulnerable to infections, which the Th1 immune system can better protect. The unique immune system changes during pregnancy necessitate an integrated multispecialty approach for managing COVID-19 positive pregnant females. The current literature reported that the outcomes of 55 COVID-19 positive pregnant females and 46 newborns did not suggest the possibility of mother-to-foetus transmission of SARS-CoV-2 infection [6]. Similarly, the International Society of Infectious Disease in Obstetrics and Gynecology (ISIDOG) guidelines also suggested no indication of vertical transmission of COVID-19 during the second or third trimester of pregnancy [7].

The rapid onset of the COVID-19 outbreak significantly impacts the medical and public health facilities. So vulnerable groups, including pregnant women, should focus on infection prevention and management through strategic preparedness and evidence-based response planning. In the previous Ebola outbreak, clinicians are often cautious about treating or providing vaccination to pregnant women considering the foetal outcomes [8]. It is crucial to ensure that women receive substantial life-saving treatment despite the threat of a contagious disease unless there is a serious clinical contraindication. Moreover, it is necessary to carefully assess the potential benefits and risks of the interventions prior to clinical-decision making to treat infected pregnant women. As surveillance systems for COVID-19 positive patients are being established, it is crucial to analyse the maternal and foetal outcomes during pregnancy.

Interestingly, in the current COVID-19 outbreak, proportionally, more men are affected than women [9]. This gender disparity can be attributed to the variability in the reporting system, susceptibility of exposure, screening and diagnosis of infection. Notably, the frequently reported symptoms of COVID-19 are cough (82%), fever (83%) and shortness of breath (31%) among hospitalised patients and elderly males with associated co-morbidities are more likely to show severe respiratory distress [10]. As there is limited published data, the possible susceptibility of pregnancy towards COVID-19 infection or the potential risk of miscarriage or foetal anomalies remains unclear.

## Methodology

### Literature Searches

The current systematic literature review was performed utilising electronic databases PubMed, MEDLINE and EMBASE. We searched for the keywords "COVID-19 AND "pregnancy" between January 1, 2020, until December 31, 2020. Due to the rapid onset of the COVID-19 pandemic, literature is scarce on this topic, so all articles were considered for analysis. Medical subject headings (MeSH) terms used were “2019-nCoV” [Title/Abstract] OR “2020-nCov” [Title/Abstract] OR “2019-20 coronavirus*” [Title/Abstract] OR “2019-2020 coronavirus*” [Title/Abstract] OR “2019 coronavirus*” [Title/Abstract] OR “coronavirus* 2019” [Title/Abstract] OR “2020 coronavirus*” [Title/Abstract] OR “coronavirus* 2020” [Title/Abstract] OR “SARS-CoV-2” [Title/Abstract] OR “SARS coronavirus 2” [Title/Abstract] OR “COVID-19” [Title/Abstract] OR “COVID-2019” [Title/Abstract] OR “coronavirus disease 2019” [Title/Abstract] OR “coronavirus disease 2020”[Title/Abstract]))) AND “Pregnancy”. All the extracted relevant articles were considered for the review analysis. All research articles describing the epidemiology, clinical characteristics and management of COVID-19 in pregnancy were included during the mentioned period.

### Inclusion/Exclusion Criteria

We included all published articles in the English language accessible on electronic databases and specific institutional sites. The retrieved articles were independently assessed and evaluated by five authors for inclusion. This process resulted in the selection of 36 articles related to clinical characteristics and management of COVID-19 in pregnancy for this review [11-46]. We have also utilised the bibliography of the retrieved articles (this process is also known as hand-searching), to look for other suitable associated articles for cross-referencing.

### Data Extraction

Databases PubMed, MEDLINE and EMBASE were initially searched thoroughly for appropriate titles. The selected titles were then screened for the abstracts and full texts. Articles that met the eligibility requirements were considered for this systematic review. Five researchers independently conducted the literature evaluation (BS, HAlH, IB, MA, and AA). The extracted data include study authors, year, Sample size Study design, Confirmed SARS-CoV-2, Vertical transmission, Maternal mortality, Neonatal mortality, Age, Gestational Age (delivery), Maternal comorbidities, Clinical features- Fever on admission Fever after childbirth, Cough, Malaise, Dyspnea, Myalgia, Sore Throat, Diarrhoea, Laboratory characteristics, CT scan, Treatment, Delivery characteristics, Maternal outcome and Neonatal outcome.

### Methodology Quality Assessment

The selected studies were assessed for methodological quality based on study design, case ascertainment, case definition, patient population, and the methodology description. We used the Joanna Briggs Institute (JBI) Critical Appraisal Checklist for Analytical Cohort studies, with 11 domains assessing, simithe larity between groups, the similarity between exposures, validity and reliability of the exposure, cofounding factors, freeness of the outcome, measurement of the outcomes, follow-up time and statistical analysis.

### Data Analysis and Synthesis

Prevalence was calculated for categorical variables. The decision to select either fixed effect or random effects model depended on the results of statistical tests for heterogeneity. Data heterogeneity was assessed using the Cochrane Q homogeneity test (significance set at p < 0.10). If the studies were statistically homogeneous, a fixed-effect model was selected. A random-effects model was used when studies were statistically heterogeneous. The Higgin’s I^2^ test is the ratio of true heterogeneity to the total variation in observed effects. A rough guide to the interpretation of the I^2^ test is 0-25%: might not be significant; 25-50%: may represent moderate heterogeneity; 50-75%: may represent substantial heterogeneity; and > 75%: considerable heterogeneity. Publication bias was visually estimated by assessing funnel plots. Pooled estimates were calculated using R 3.5.1 software.

## Results

Out of 4005 records that were identified, 476 duplicates were removed. One hundred eighty-nine records were screened, reviews, letters, news articles, patient-education handouts, retracted papers were excluded. Finally, 36 original studies were included in this systematic review.

**Figure 1:**
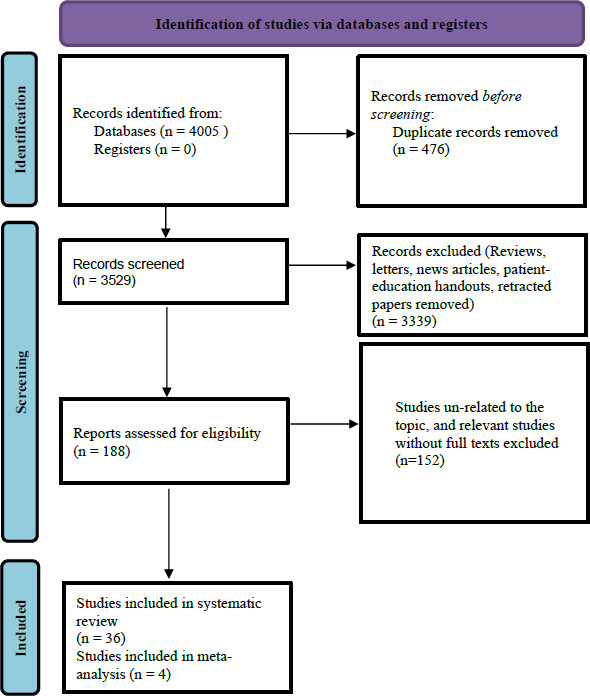
The PRISMA flow diagram for the systematic Review and Metaanalysis

We used the PRISMA diagram to illustrate the flow of information through the different phases of the systematic review, it maps out the number of records identified, included and excluded (Figure 1).

### Quality assessment

**Figure 2:**
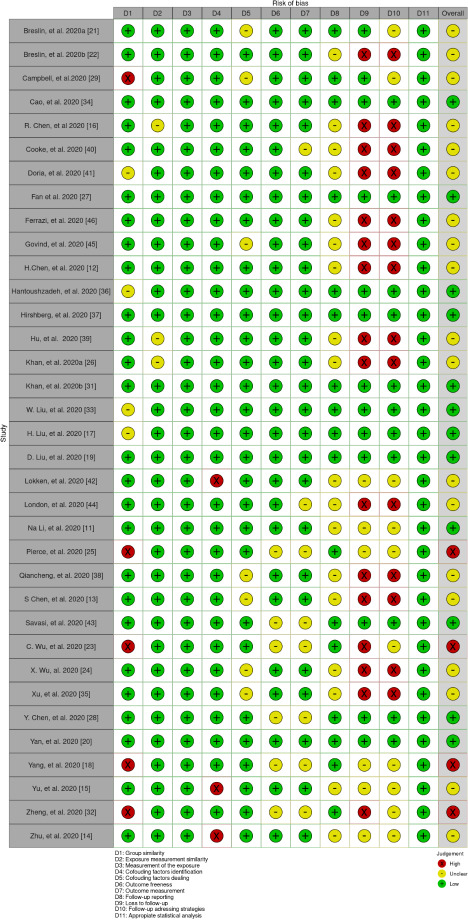
Risk of Bias

**Figure 3:**
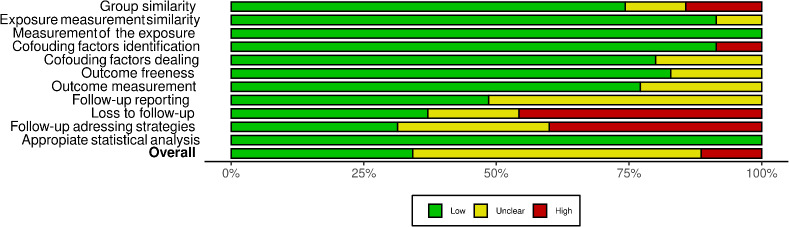
Risk of bias summary

The risk of bias of included studies was assessed, for figure elaboration and analysis we implemented Risk-of-bias VISualization (robvis): An R package and Shiny web app for visualizing risk-of-bias assessments as shown in Figures 2 and 3. Overall quality was low in the studies found; nevertheless, the domains of follow-up time, loss to follow up and strategies to address incomplete follow up were deficient in most of the studies because of no clear description or a statement in this regard.

Table 1 depicts the characteristics of studies included in this systematic review. Out of 36 original studies, 22 studies were conducted in China, nine in the USA (United States of America), two in the UK (United Kingdom), and one each in Italy, Portugal, and Iran. Two studies were case-control [11, 18], one was retrospective prospective study [25], two were prospective [32, 33], the remaining 31 studies were retrospective [12-17, 19-24, 26-31, 34-46]. The sample size and confirmed COVID-19 (%) are shown in Table 1.

Table 2 determines the maternal characteristics, consisting of maternal age and gestational age during delivery. The age of the patient was raging from 19-41 and gestational age was 31-41 weeks. Table 3 depicts the mode of delivery comprised of caesarean section, preterm delivery, vaginal delivery and the total number of deliveries. The caesarean section was the preferred method of delivery in a study conducted by Na Li et al. [11]. The procedure was conducted in 87.5% of confirmed cases and 88.9% in suspected cases. In all 100% of cases, caesarean section was performed in the studies conducted by H.Chen et al., Yu et al., Chen et al., Fan et al., Khan et al., Hirshberg et al., and Cooke et al [12, 15, 16, 27, 31, 37, 40],. Vaginal delivery was the least preferred mode of delivery in all the selected studies. In a study conducted by Pierce-Williams et al., 88% were preterm delivery in severe COVID-19 patients.

Table 4 shows the clinical features of pregnant women due to COVID-19 viz. fever, cough, malaise, dyspnoea, myalgia, sore throat. The studies conducted by Khan et al. and Zheng et al. showed fever on admission in 66.7% and 73% cases [14,26]. Cough was present in 100% of patients in the study conducted by Khan et al [26]. In 60% of the cases in a study conducted by D. Liu et al. [19] myalgia and sore throat were not common clinical features.

Table 5 elicits the various laboratory findings, including lymphocytopenia, elevated CRP concentration, and confirmed SARS- Cov -2 cases. Table 6 shows the CT Scan findings of the patients.

Various pharmacological approaches of the patients are depicted in Table 7. Antibiotics were prescribed in most of the patients. In a study conducted by Na Li et al. 100% of confirmed and suspected cases received antibiotics [11]. Similarly in studies conducted by H.Chen et al. 100%, Yu et al. 100%, D. Liu et al. 100%, C.Wu et al. 100%, Khan et al. 100%, Fan et al. 100%, Xu et al. 100%, and Hantoushzadeh et al. 100% patients received antibiotics therapy [12, 15, 19, 23, 26, 27, 35, 36]. Other modes of therapy were antiviral drugs, which were prescribed in 67% of cases in a study conducted by H. Chen al. and in 100% cases in the studies conducted by Yu et al., Hantoushzadeh et al., Hirshberg et al., Xu et al. and Khan et al. respectively[12, 15, 26, 36, 37, 35, 31]. High-flow 02 was given in 100% cases in studies conducted by H. Chen et al. and Yu et al. [12, 15]. Table 8 shows maternal comorbidities and maternal outcomes. A study conducted by Hantoushzadeh et al. reported 77% maternal mortality and a maternal ICU (Intensive Care Unit) admission of 77% Cooke et al. showed a maternal ICU admission in 100% of cases. In comparison, other studies included in this systematic review did not report high percentages of maternal ICU admission.

Table 9 shows the neonatal outcomes which varied a lot: 1% of neonatal death was reported by Yan et al., 36% by Hantoushzadeh et al., 13% by Lokken et al. and 2% by London et al., respectively [20, 36, 42, 44]. Other neonatal outcomes such as Intrauterine Fetal Death, Vertical Transmission, Intrauterine Fetal Distress, Mean Birth Weight, Low Birth Weight, APGAR score at 1 minute and five after birth is shown in Table 8.

### Outcome measures

**Figure 4:**
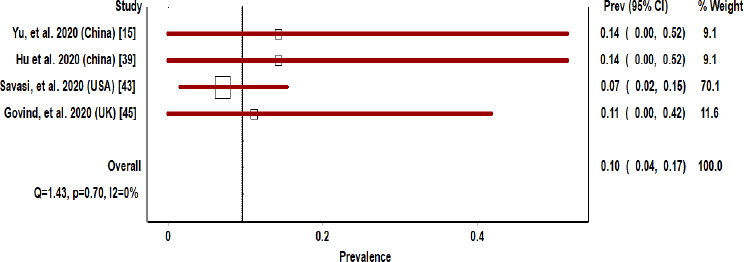
Forest plot of pregnant COVID 19 patients based on their vertical transmission

Figure 4 depicts the meta-analysis of the four studies of pregnant COVID-19 patients based on their vertical transmission. Pooled prevalence of vertical transmission was 10%, 95% CI: 4-17% (Figure 4).

**Figure 5:**
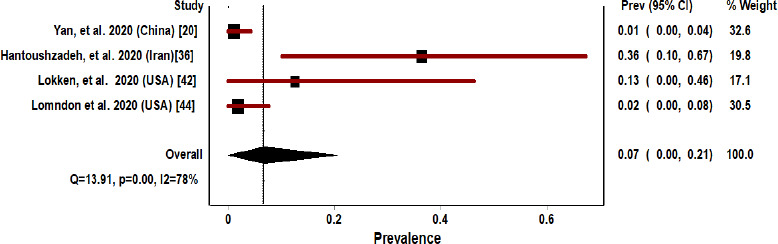
Forest plot pregnant COVID 19 patients based on their neonatal mortality

Figure 5 depicts the meta-analysis of the four pregnant COVID-19 patient studies based on neonatal mortality. Pooled prevalence of neonatal mortality was 7%, 95% CI: 0-21% (Figure 5).

### Heterogeneity among included studies

The results for the heterogeneity test for the meta-analysis of the vertical transmission and neonatal mortality among pregnant COVID 19 patients are displayed towards the bottom of the Forest plot in the line. For vertical transmission group, Q [χ^2^]=1.43, P=0.70, I^2^=0%, (Figure 4). However, I^2^ was<25%, a fixed-effect model was considered for vertical transmission. For the neonatal mortality group, Q [χ^2^] =13.91, P=0.001, I^2^=78%, tau2=0.109 (Figure 5). However, I^2^ was>25%, a random-effect model was considered for neonatal mortality.

### Publication bias and funnel plots

**Figure 6:**
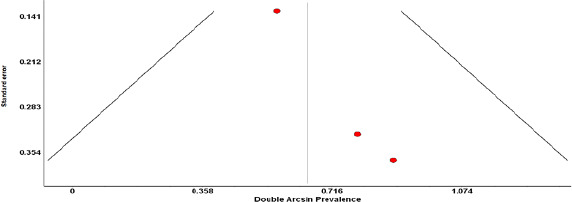
Funnel plot of pregnant COVID 19 patients based on their vertical transmission

**Figure 7.**
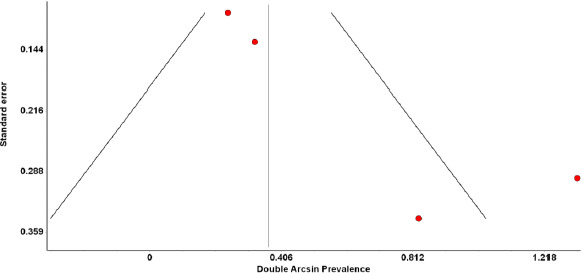
Funnel plot of COVID-19 pregnant patients based on their neonatal mortality

For all of the above analyses, sensitivity analysis yielded consistent results. Based on a visual inspection of the funnel plot, there was evidence of publication bias for the included studies (Figure 6 and 7). The funnel plots exhibited studies with sizeable standard error and were not symmetrical.

## Discussion

### Clinical Manifestations and Complications during Pregnancy

Based on the earlier evidence of Middle East Respiratory Syndrome (MERS) and Severe Acute Respiratory Syndrome (SARS) the clinical manifestation of these infections during pregnancy varies from asymptomatic presentation to severe respiratory illness and mortality. A recent retrospective study presented the clinical profile of nine pregnant women diagnosed with COVID-19 during the third trimester. The authors reported that the clinical presentation of COVID-19 positive pregnant women was comparable with the non-pregnant female patients, and the frequent symptoms were fever (78%), cough (44%), myalgia (33%), sore throat and malaise (22% each). Lymphopenia was reported in five patients and none of them had developed severe pneumonia that requires mechanical ventilation and all survived. All women had a caesarean section and the Apgar score was 8-9 at 1 minute and 9-10 at 5 minutes. Four out of nine deliveries had pre-term labour but after 36 gestational weeks and seven patients developed pregnancy related-complications [12].

Another series of nine COVID-19 positive pregnant women with ten infants (one had twins) by Zhu et al., reported onset of symptomatic presentation prior to delivery (1-6 days) in four, on the day of delivery in two, and post-delivery (1-3 days) in three cases [14]. Similar to the earlier report, the clinical presentation of COVID-19 cases was comparable to non-pregnant patients. Of the nine pregnancies, six patients had intrauterine foetal distress, seven had a caesarean section, six were preterm deliveries and four had pregnancy-related complications [14].

Yu et al. studied seven pregnant COVID-19 patients with a mean age of 32 (range 29–34 years) admitted during early 2020 [15]. The average gestational age was over 39 weeks. Six patients had a fever as their chief clinical manifestation, shortness of breath, and cough and diarrhoea in one patient each. Caesarean sections were performed in all patients within three days of clinical presentation. All women had good maternal and neonatal outcomes but one neonate was tested positive for COVID-19 36 hours after birth [15]. Wu et al. reported eight pregnant patients aged 26 to 35 years [24]. Six of these women were laboratory-confirmed and two were highly suspected SARSCoV-2 infection cases. Six had caesarean sections because of premature rupture of membrane, preeclampsia, fetal distress, or the history of caesarean section. Notably, intensive care or mechanical ventilation was not required in any patients. Four out of eight pregnant women remained asymptomatic prior to delivery, but later developed symptoms post-partum [24]. Breslin et al. reported seven confirmed COVID-19 cases in pregnancy in the USA, presented with symptoms such as cough, fevers, myalgia, chest pain, and headache. Two patients required supportive care with intravenous hydration [22]. Another study from the USA described 43 patients, 14 (32.6%) were asymptomatic, later on, ten patients developed symptoms during the hospital course or early after postpartum discharge [21]. Three required antenatal admission for viral symptoms of the other 29 (67.4%) symptomatic patients. The disease was mild in 86%, severe in 9.3%, and critical in 4.7%. Wu et al. demonstrated a significant difference in the median time for hospitalisation among asymptomatic and symptomatic patients (14 vs. 25.5 days, p<0.05) [24]. This study was conducted retrospectively among 23 pregnant patients diagnosed with COVID-19 and admitted to the hospital in Wuhan [24].

Chen and colleagues [13] reported five young pregnant COVID-19 patients aged 25 to 31 years, gestational age from 38 to 41 weeks, who were admitted to the hospital. The women developed low-grade fever within 24 hours post-partum. The renal and liver function profile was unremarkable for all patients. CT scan images revealed abnormal bilateral ground-glass opacity (three cases) or unilateral lobe (two cases). Three had normal births and two underwent caesarean sections; all labours were smoothly processed with no maternal or newborn complications. The Apgar scores were 10 points at 1 and 5 minutes after delivery [13]. Liu et al. reported clinical differences among pregnant and non-pregnant. The laboratory-confirmed and clinically diagnosed COVID-19 pregnant women had higher initial body temperature, leukocytosis and elevated neutrophil ratio, and lymphopenia as compared to laboratory-confirmed non-pregnant patients [17]. The CT imaging revealed lesions with peripheral (98%) and bilateral (67%) distributions. For the non-pregnant adults, pure ground-glass opacity (72%) was the most frequent finding [17].

A retrospective study by Zhang et al. compared the pregnancy outcomes among COVID-19 positive and negative women having similar gestational age and delivered by caesarean section [49]. The authors suggested no additional risk of intra-operative blood loss during delivery with a comparable birth weight in both groups. In addition, the two groups were comparable for meconium-stained amniotic fluid, foetal distress, preterm birth, and neonatal asphyxia between COVID-19 patients and controls. Based on ten neonates in this study, there was no evidence of mother-to-fetal vertical transmission of COVID-19 infection [49]. Yang et al. tested the throat swab samples within 24 h after birth for COVID-19 which revealed no evidence of SARS-CoV-2 transmission from infected mother to newborn babies [18].

A case-control study conducted by Li et al. [48] included 16 pregnant women positive for COVID-19 and 18 suspected cases in the third trimester admitted to the hospital. Notably, two patients were presented with respiratory symptoms (fever and cough) on admission. Most of the patients had chest CT (computed tomography) scan images suggesting typical COVID-19 pneumonia. Similar to other studies, these patients had lower white blood cells and neutrophils counts, with deranged alanine aminotransferase (ALT) and C-reactive protein (CRP) levels on admission compared to controls. Postpartum blood investigations revealed increased eosinophils, white blood cells, neutrophils and CRP. Three women with confirmed COVID-19 and three in the control group developed pneumonia and had preterm delivery secondary to maternal complications. None of the subjects had a respiratory failure in the hospital, all newborns were negative for infection, and none had severe neonatal complications [48].

Zaigham et al. conducted a systematic review to evaluate the maternal and perinatal outcomes in pregnancies with COVID-19 [50]. The authors included all case reports and series from December 8, 2019 and April 1, 2020 and identified a total of 108 pregnancies. During their third trimester of pregnancy, most studies observed women mostly presented with fever (68%) and coughing (34%). Lymphocytopenia (59%) with elevated CRP (70%) was also common. Most (91%) deliveries were by caesarean section and three pregnant women required ICU admissions. Overall, there was one neonatal and intrauterine death with no maternal mortality [50]. The authors concluded that most pregnant women were discharged without significant complications, but there are some cases with severe maternal morbidity and perinatal deaths secondary to COVID-19 infection. Also, vertical transmission of the COVID-19 infection from mother to foetus could not be ruled out [50].

The emerging evidence also indicates that vertical transmission of SARS-COV-2 infection is unlikely, but still considering the smaller proportion of pregnant cases, the mode of transmission of COVID-19 needs further exploration. Two published reports have evidence of IgM antibodies for SARS-COV-2 in neonatal serum after birth [23,51]. Since, IgM antibodies are not known to cross the placenta, the presence of these antibodies indicates the neonatal immune response to maternal in-utero infection. Although conflicting results were published in previous case reports from China, we must remember that case reports are the lowest form of evidence in the hierarchy of evidence [52]. These reports found that the samples of amniotic fluid, placenta and neonatal throat swabs, cord blood, genital fluid, and breast milk were negative for COVID-19 infection, indicating no vertical transmission evidence [12, 14, 29, 48]. This also suggests that breastfeeding should be encouraged if at all possible, with precautions such as washing hands before holding the baby and the mother wearing a face mask during breastfeeding.

Previous studies have reported that SARS-CoV-2 has more genetic proximity (88% sequence homology) with two SARS-like coronaviruses derived from bat i.e. bat-SL-CoVZC45 and bat-SL-CoVZXC21, as compared to SARS-CoV-1 (79% sequence identity) and MERS-CoV (about 50% identity). However, modelling studies using bioinformatics tools showed that receptor binding domain structure of SARS-CoV-2 and SARS-CoV-1 are similar, which suggests a similar course of pathogenesis among the two infections [53-55]. Hence, there is a lower possibility of vertical transmission of COVID-19 infection similar to that of SARS-CoV-1. The pooled clinical records of 116 pregnant women with COVID-19 from China concluded no evidence of SARS-CoV-2 vertical transmission of infection among pregnant women during the third-trimester [20].

## Clinical Management

Pregnant women are considered high risk, so those with suspicion of COVID-19 must be isolated, clinically assessed, and investigated for the infection [56]. Zeng et al. reported that all six COVID-19 pregnant women in their study had caesarean deliveries in their third trimester in negative pressure isolation rooms [23]. All mothers should wear masks and medical staff should use protective suits and double masks. The infants were isolated immediately post delivery, and throat swabs and blood specimens were found to be found negative by RT-PCR test [23]. The COVID-19 Obstetrics Task Force, from Italy stressed the need to isolate newborns from COVID-19-positive and symptomatic mothers and use pumps to express breast milk [47].

Pregnant patients who tested positive for infection must be immediately shifted to an isolation ward, ideally an isolation ward in a tertiary care hospital equipped with appropriate medical facilities and multi-disciplinary team to manage severely ill pregnant women. Such patients should be triaged and clinically evaluated for risk stratification from the beginning. Patients with mild symptoms often have stable vital signs, while those with severe pulmonary distress have respiratory rate ≥30/min, resting oxygen saturation ≤93%, and the ratio of arterial blood oxygen partial pressure (PaO2)/oxygen concentration (FiO2) to be ≤300 mmHg [7]. Importantly, those with critical illness may develop shock with organ dysfunction, a respiratory failure requiring mechanical ventilation, extra-corporal membrane oxygenation (ECMO) to manage refractory hypoxemia [57]. Universal COVID-19 testing for all pregnant women closer delivery time will help in triaging for hospital isolation, bed assignment, personal protective equipment (PPE) by healthcare providers and improved neonatal care [58].

A multispecialty team of obstetricians, midwives, intensivists, microbiologists, anaesthetists and neonatologist should be available to manage pregnant women with COVID-19 primarily to manage severe and critical cases [59]. Importantly, physiological adaptations in pregnancy should be given higher consideration while treating pregnant females with confirmed diagnosis of COVID-19 infection. For appropriate patient care and infection prevention among healthcare workers, the clinical staff involved in the care of COVID-19 patients should properly use PPE such as N95 masks, gowns, gloves, and goggles [60]. However, we need to remember that all pregnant women, irrespective of COVID-19 infections, deserve the best quality of care pre and post-partum, should be treated with respect and dignity; and have an attendant of choice available during delivery [61].

Pregnant women with infection should rest, and be closely monitored for adequate nutritional support, hydration, electrolyte balance, vital signs and oxygen saturation to maintain good physiological status [56]. In patients with acute respiratory distress, supplemental oxygen through a high-flow nasal cannula should be inspired with 60%-100% concentration at a flow rate of 40 L/min, depending upon the severity of hypoxemia [62]. In critically ill patients, mechanical ventilatory support or extra-corporal membrane oxygenation should be considered to maintain oxygen saturation. The other complications more common in COVID-19 patients may include sepsis and septic shock, acute kidney injury, and virus-induced cardiac injury [63]. Therefore, in severe cases it is crucial to closely monitor the derangement of arterial blood gases, serum lactate, renal and liver function profile and cardiac enzymes as clinical indicators of physiologic deterioration.

In China, the antiviral treatment has been administered routinely to manage COVID-19 patients which some recommended even for pregnant patients [56]. Particularly, combination therapy of antiproteases namely Lopinavir/Ritonavir is preferred and considered a relatively safer drug regimen in pregnant COVID-19 patients. It is recommended to have oral administration of Lopinavir/ Ritonavir (200 mg/50 mg per capsule) two capsules orally twice a day along with the inhalation of nebulised α-interferon (5 million IU in 2 mL of sterile water for injection) twice daily [64]. The World Health Organization (WHO) suggested taking precautions and judicious risk-benefit assessment prior to administering of any therapeutic drug among pregnant females except for clinical trials. Interestingly, Remdesivir a nucleotide analogue, and chloroquine/hydroxychloroquine, a known antimalarial drug, are emerging potential therapeutic agents against COVID-19 which inhibit the SARS-COV-2 virus replication in in-vitro studies [65]. In-vitro studies demonstrated inhibition activity of Chloroquine against SARS-COV-2 virus and potentially minimised the duration of viral shedding. However, we are waiting for the outcomes of various clinical trials to assess the therapeutic implication of these drugs in human subjects. More recently the ISIDOG guidelines reminded us that anti-retrovirals are not indicated in pregnancy unless there are no other treatment options available to manage severe illness [7]

The SARS-CoV-2 virus is known to cause extensive pulmonary damage that eventually enhances the possibility of secondary bacterial pneumonia [66]. Therefore, the administration of antibiotics prophylaxis is primarily recommended, if there is a diagnosis of secondary bacterial infection. Moreover, antibiotic treatment should be initiated immediately if bacterial sepsis is suspected [62]. Mainly, intravenous administration of Ceftriaxone can be initiated even before the availability of culture and sensitivity results.

To date, there is no available standard treatment for COVID-19, so novel or off-labelled drug should be considered experimental, and a detailed explanation has to be provided to the patients and partner prior to administration. For instance, Corticosteroids can be used to prevent neonatal lung hypoplasia, necrotic enterocolitis and interventricular haemorrhage due to prematurity. Although, (Hydro) chloroquine is considered a safe drug for pregnant women, all precautions must be considered before administration in COVID-19 patients.

### Prevention

Evidence suggested that the incubation period of COVID19 ranges from 2-14 days, but the infected individual can also transmit the virus despite being asymptomatic during this period. Therefore, pregnant women should avoid public transport, crowds, contact with sick people, unnecessary travel, maintain appropriate social and personal hygiene, including proper washing of hands as per recommendation [20]. Moreover, pregnant women with symptoms of cough, fever, myalgia, fatigue, sore throat or shortness of breath should seek immediate medical consultation. Women with a clinical suspicion of COVID-19 infection and travel history must be isolated and investigated [67]. Also, there is a need to provide professional psychological support to overcome mental health ilness among pregnant women at risk of developing severe anxiety and depression [68].

As per the current guidelines of the UK’s Royal College of Obstetricians and Gynaecologists (RCOG), the Royal College of Midwives (RCM), and the Royal College of Paediatrics and Child Health (RCPCH), health workers exceeding 28 weeks of pregnancy must avoid direct patients contact whether they are infected with COVID-19 or not [69, 70]. However, healthcare professionals with less than 28 weeks of pregnancy, can directly deal with the patients using appropriate PPEs. It is advisable to have a more precautionary approach for pregnant women completed 28 weeks’ gestation, or those with underlying co-mobidities such as heart or lung diseases woking in clinical services. If possible, such females should work from home, stay away from infected individuals, and avoid unnecessary social contact, Moreover, under these circumstances the healthcare workers have opportunities for flexible working capacity and newer approaches such as telephone or video conference consultations, or assigned administrative duties [70].

With this outbreak, there is a realisation to strengthen our healthcare capacity to tackle the emergent novel outbreaks, propose stringent regulations to contain the spread of infectious diseases and implement public education among families, communities and other religious groups to avoid further outbreak and preparedness though transparency and solidarity. In order to avoid delayed responses to an outbreak by the administration, timely reporting of emergent infectious agents is of paramount importance [71]. Proper infection control and preventive measures should be implemented in the healthcare facilities through isolation of confirmed cases and recommended PPE to protect medical professionals and other healthcare staff from exposure to communicable diseases [72].

It is worth noting that this extensive systematic review only cover papers published in 2020, and hence studies conducted in or before 2020. This was before the emergence of variants of COVID-19, especially the delta and omicron variants.

## Conclusion

The COVID-19 outbreak is exponentially increasing worldwide. A better understanding of the disease transmission, major risk factors, vulnerable population, and the outcome is crucial for prevention and management. At present, limited information is available for pregnant women with COVID-19 to propose best practices for specialised care. However, the current literature suggests that pregnant women with COVID-19 infection may develop severe clinical manifestations. Therefore, surveillance systems for COVID-19 positive patients are being established to analyse the maternal and foetal outcomes during pregnancy. Also, awareness about the management of COVID-19 positive pregnancies and prevention of neonatal infection is vital portant for healthcare professionals. Proper vigilance and monitoring of the disease spread and rapid implementation of preventive measures are crucial to containing the spread of infection in the community. The ready availability of standard respiratory support to manage severe COVID-19 infection in pregnancy is crucial and should be implemented rigorously by a multidisciplinary team. For better understanding it is essential to have systematic data reporting for evidence based clinical assessment, management, and pregnancy outcomes in infected females that will guide the healthcare facilities with limited resources to manage the vulnerable population in an outbreak.

## Figures and Tables

**Table 1:** Characteristics of studies included in the systematic review

Author, year (Country)	Sample size (N)	Study design	Confirmed SARS-CoV-2 (%)
Na Li, et al. 2020 (China) [11]	276	Retrospective case-control	16/276 (6.0)
H.Chen, et al. 2020 (China) [12]	9	Retrospective	9/9(100)
S. Chen. et al. 2020 (China) [13]	5	Retrospective	5/5(100)
Zhu et al. 2020 (China) [14]	9	Retrospective	9/9(100)
Yu, et al. 2020 (China) [15]	7	Retrospective	7/7(100)
R.Chen, et al. 2020 (China) [16]	17	Retrospective	17/17(100)
H.Liu, et al. 2020 (China) [17]	41	Retrospective	41/41(100)
Yang, et al. 2020 (China) [18]	55	Retrospective case-control	13/55(24)
D.Liu, et al. 2020 (China) [19]	15	Retrospective	15/15(100)
Yan, et al. 2020 (China) [20]	116	Retrospective	65/116(56.0)
Breslin, et al. 2020a (USA) [21]	43	Retrospective	43/43(100)
Breslin, et al. 2020b (USA) [22]	7	Retrospective	7/7(100)
C.Wu, et al. 2020 (China) [23]	8	Retrospective	8/8(100)
X.Wu, et al. 2020 (China) [24]	23	Retrospective	23/23(100)
Pierce-Williams, et al. 2020 (USA) [25]	64	Multicenter Cohort Retrospective-Prospective	64/64(100)
Khan, et al. 2020a (China) [26]	3	Retrospective	3/3(100)
Fan, et al. 2020 (China) [27]	2	Retrospective	2/2(100)
Y. Chen, et al. 2020 (China) [28]	4	Retrospective	4/4(100)
Campbell, et al.2020 (USA) [29]	30	Retrospective	30/30(100)
Baergen, et al. 2020 (USA) [30]	20	Retrospective	20/20(100)
Khan, et al. 2020b (China) [31]	17	Retrospective	17/17(100)
Zheng, et al. 2020 (China) [32]	33	Prospective	33/33(100)
W.Liu, et al. 2020 (China) [33]	19	Prospective	19/19(100)
Cao, et al. 2020 (China) [34]	10	Retrospective	10/10(100)
Xu, et al. 2020 (China) [35]	5	Retrospective	5/5(100)
Hantoushzadeh, et al. 2020 (Iran)[36]	9	Retrospective	9/9(100)
Hirshberg, et al. 2020 (USA) [37]	5	Retrospective	5/5(100)
Qiancheng, et al. 2020 (China) [38]	28	Retrospective	28/28(100)
Hu, et al. 2020 (China) [39]	7	Retrospective	7/7(100)
Cooke, et al. 2020 (UK) [40]	2	Retrospective	2/2(100)
Doria, et al. 2020 (Portugal) [41]	12	Retrospective	12/12(100)
Lokken, et al. 2020 (USA) [42]	46	Retrospective	46/46(100)
Savasi, et al. 2020 (USA) [43]	77	Retrospective	77/77(100)
London, et al. 2020 (USA) [44]	68	Retrospective	68/68(100)
Govind, et al. 2020 (UK) [45]	9	Retrospective	9/9(100)
Ferrazi, et al. 2020 (Italy) [46]	42	Retrospective	42/42(100)

**Table 2:** Maternal characteristics

Authors	Age	Gestational Age (delivery)
Na Li et al. 2020 [11]		
Confirmed Case (16)	30.9 ±3.2	38±0.2
Suspected Case (18)	29.8 ± 2.3	38±2. 9
Control 2020(121)	30.1 ± 3.3	39±0.7
Control 2019(121)	29.3 ± 2.6	38±6.9
H.Chen, et al. 2020 [12]	29.8 years	36-39 wks + 4 days
S.Chen. et al. 2020 [13]	29 years	38-41 weeks
Zhu et al. 2020 [14]	31 years	31-39 weeks
Yu, et al. 2020 [15]	32 years	39 weeks + 1 day
R.Chen, et al. 2020 [16]	29.1 years	>37 weeks
H.Liu, et al. 2020 [17]	31 years	-
Yang, et al. 2020 [18]		
Confirmed (13)	30.2 ± 2.3	38.2 ± 2.3
Control (42)	29.8 ± 3.4	38.8 ± 1.8
D.Liu, et al. 2020 [19]	32 ± 5	32 ± 8
Yan, et al. 2020 [20]	30.8 ± 3.8	38.0 (36.0,39.1)
Breslin, et al. 2020a [21]	29.7 ± 6.0	37 weeks
Breslin, et al. 2020b [22]	33.8 years	33 weeks+3 days
C.Wu, et al. 2020 [23]	29.8	33.3
X.Wu, et al. 2020 [24]	29	-
Pierce-Williams, et al. 2020 [25]		
Severe (44)	32.0±6.0	37.7±1.6
Critical (20)	35.9±4.3	31.9±3.8
Khan,et al. 2020a [26]	29.3	38 weeks
Fan, et al. 2020 [27]	31.5	36-38
Y.Chen, et al. 2020 [28]	29	37-39 weeks
Campbell, et al. 2020 [29]	30-35	37 weeks
Baergen, et al. 2020 [30]	19-41	32-40 weeks
Khan, et al. 2020b [31]	37.82	38.1
W.Liu, et al. 2020 [33]	31	38.6±1.5
Cao, et al. 2020 [34]	29-35	33-40
Xu, et al. 2020 [35]	29	36.4±3
Hantoushzadeh, et al. 2020 [36]	32	24-38
Hirshberg, et al. 2020 [37]	33	25-31
Qiancheng, et al. 2020 [38]	30 (26.75–32)	38 (36.5–39)
Hu, et al. 2020 [39]	33	37-41
Cooke, et al. 2020 [40]	34	28
Doria, et al. 2020 [41]	32	30-41
Lokken, et al. 2020 [42]	29 (26-34)	27 weeks
Savasi, et al. 2020 [43]	32	37
London, et al. 2020 [44]	30 years	65/68 in 3rd tri
Govind, et al. 2020 [45]	31 (18-39)	39 weeks
Ferrazi, et al. 2020 [46]	21-44	30 weeks

**Table 3:** Delivery characteristics

Authors	Total Number of Deliveries	Pt. not delivered during time of reporting	Cesarean delivery	Pre-term	Vaginal
Na Li et al. 2020[11]					
Confirmed Case (16)	152/276 (55.0)	124/276 (45.0)	14/16(87.5)	3 /16 (18.8)	2/16(12.5)
Suspected Case (18)			16/18 (88.9)	3/18 (16.7)	-
Control 2020(121)			57/121 (47.1)	7/121 (5.8)	-
Control 2019(121)			44 /121(36.4)	6/121 (5.0)	-
H.Chen, et al. 2020 [12]	9/9(100)	-	9/9(100)	4/9(44)	-
S.Chen. et al. 2020 [13]	5/5(100)	-	2/5(40)	-	3/5(60)
Zhu et al. 2020 [14]	9/9(100)	-	7/9(78)	5/9(56)	2/9(22)
Yu, et al. 2020 [15]	7/7(100)	-	7/7(100)	-	-
R.Chen, et al. 2020 [16]	17/17(100)	-	17/17(100)	3/17(18)	-
H.Liu, et al. 2020 [17]	16/41(39)	25/41(61)	-	-	-
Yang, et al. 2020 [18]					
Confirmed (13)	55/55(100)	-	9/13(69.2)	-	4/13(30.8)
Control (42)			30/42(71.4)	-	12/13(28.6)
D.Liu, et al. 2020 [19]	11/15(73)	4/15(27)	10/11(91)	-	1/11(9)
Yan, et al. 2020 [20]	99/116 (85.3)	17/116 (14.6)	85/99 (85.9)	21/99 (21.2)	14/99(14.1)
Breslin, et al. 2020a [21]	18/43 (41.9)	25/43(58.1)	8/18(44.4)	-	10/18(55.5)
Breslin, et al. 2020b [22]	-	-	-	-	-
C.Wu, et al. 2020 [23]	8/8(100)	-	6/8(75)	1/8 (12.5)	2/8(25)
X.Wu, et al. 2020 [24]	20/23 (86.9)	-	18/20(90)	-	2/20(10)
Pierce-Williams, et al. 2020 [25]					
Severe (44)	15/44(34)	29/44(66)	8/15 (53%)	4 /15 (27%)	7/15 (47%)
Critical (20)	17/20(85)	3/20(15)	16/17 (94%)	15/17 (88%)	1/17 (6%)
Khan, et al. 2020a [26]	3/3(100)	-	-	1/3 (33.3)	3/3(100)
Fan et al. 2020 [27]	2/2(100)	-	2/2(100)	-	-
Y.Chen, et al. 2020 [28]	4/4(100)	-	3/4(75)	-	1/4(25)
Campbell, et al.2020 [29]	10/30(33)	20/30(66)	10/30(33)	-	-
Baergen, et al. 2020 [30]	17/20(85)	-	5/20(25)	-	15/20(75)
Khan, et al. 2020b [31]	17/17(100)	-	17/17(100)	5/17 (29.4)	-
Zheng, et al. 2020 [32]	33/33(100)	-	27/33(81)	4/33(12)	-
W.Liu, et al. 2020 [33]	19/19(100)	-	18/19(95)	-	1/19(5)
Cao, et al. 2020 [34]	10/10(100)	-	8/10(80)	3/10(30)	2/10(20)
Xu, et al. 2020 [35]	10/10(100)	-	4/5(80)	2/5(40)	1/5(20)
Hantoushzadeh, et al. 2020 [36]	11/11(100)	2/11(18)	8/11(73)	4/11(36)	1/11 (9.1)
Hirshberg, et al. 2020 [37]	3/5(60)	2/5(40)	3/3(100)	3/3(100)	-
Qiancheng, et al. 2020 [38]	23/28(82)	5/28(18)	17/23(74)	01/23(4)	5/23(22)
Hu, et al. 2020 [39]	7/7(100)	-	6/7(86)	-	1/7(14)
Cooke, et al. 2020 [40]	2/2(100)	-	2/2(100)	2/2(100)	-
Doria, et al. 2020 [41]	10/12(83)	2/12(17)	6/10(60)	-	4/10(40)
Lokken, et al. 2020 [42]	8/46(17)	-	3/8 (37.5)	1/8 (12.5)	5/8 (62.5)
Savasi, et al. 2020 [43]	57/77(74)	20/77(26)	34/57(61)	12/57(21)	23/57(39)
London, et al. 2020 [44]	55/67(82)	12/67(18)	22/55(40)	12/55(22)	33/55(60)
Govind, et al. 2020 [45]	9/9(100)	-	8/9(89)	2/9(22)	1/9(11)
Ferrazi, et al. 2020 [46]	42/42(100)	-	18/42(43)	11/42 (26.2)	24/42(57)

**Table 4:** Presenting signs and symptoms

Authors	Fever on admission	Fever after childbirth	Cough	Malaise	Dyspnea	Myalgia	Sore Throat	Diarrhea
Na Li et al. 2020 [11]								
Confirmed Case (16)	4/16 (25.0)	1/16 (5.6)	-	-	-	-	-	-
Suspected Case (18)	1 /18(5.6)	6/18 (33.3)	1/18 (5.6)	-	1/18 (5.6)	-	1 /18(5.6)	-
Control 2020(121)	-	-	-	-	-	-	-	-
Control 2019(121)	-	-	-	-	-	-	-	-
S.Chen. et al. 2020 [13]	-	5/5(100)	1/5(20)	-	-	-	-	-
Zhu et al. 2020 [14]	4/9(44)	2/9(22)	4/9(44)	-	-	-	1/9(11)	1/9(11)
Yu, et al. 2020 [15]	6/7(86)	-	1/7(14)	-	1/7(14)	-	-	-
R.Chen, et al. 2020 [16]	4/17(24)	-	4/17(24)	1/17(6)	1/17(6)	-	1/17(6)	-
H.Liu, et al. 2020 [17]	16/41(39)	14/41(34)	15/41(37)	5/41(12)	5/41(12)	-	-	-
Yang, et al. 2020 [18]								
Confirmed (13)	2/13(15.4)	8/13(61.5)	-	-	-	-	-	-
Control (42)	11/42(26.2)	20/42(47.6)	-	-	-	-	-	-
D.Liu, et al. 2020 [19]	13/15(87)	1/11(9)	9/15(60)	4/15(27)	1/15(7)	3/15(20)	1/15(7)	1/15(7)
Yan, et al. 2020 [20]	59/116(50.9)	-	33/116 (28.4)	15/116 (12.9)	3/116 (2.6)	6 /116 (5.2)	10/116 (8.6)	1 /116 (0.9)
Breslin, et al. 2020a [21]	14/29 (48.3)	-	19/29 (65.5)	11/29(38)	7/29(24.1)	-	-	-
Breslin, et al. 2020b [22]	2/7 (28.5)	-	3/7 (43.0)	-	-	3/7(43.0)	-	-
C.Wu, et al. 2020 [23]	1/8(12.5)	3/8(37.5)	-	-	-	-	-	-
X.Wu, et al. 2020 [24]	4/23(17.4)	-	6/23(26.1)	-	-	-	-	-
Pierce-Williams, et al. 2020 [25]								
Severe (44)	-	-	-	-	-	-	-	-
Critical (20)	-	-	-	-	-	-	-	-
Khan, et al. 2020a [26]	2/3 (66.6)	-	3/3(100)	-	1/3 (33.3)	-	-	-
Fan, et al. 2020 [27]	1/2(50)	-	-	-	1/2(50)	-	-	1/2(50)
Y.Chen, et al. 2020 [28]	3/4(75)	-	2/4(50)	2/4(50)	2/4(50)	-	-	-
Campbell, et al.2020 [29]	-	-	-	-	-	-	-	-
Baergen, et al. 2020 [30]	10/20(50)	-	-	-	-	-	-	-
Khan, et al. 2020b [31]	3/17(18)	-	6/17(35)	-	2/17(12)	-	-	3/17(18)
Zheng, et al. 2020 [32]	24/33(73)	15/33(45)	30/33(91)	-	-	-	-	-
W.Liu, et al. 2020 [33]	11/19(58)	-	5/19(26)	-	-	-	-	-
Cao, et al. 2020 [34]	2/10(20)	5/10(50)	1/10(10)	1/10(10)	-	-	-	-
Xu, et al. 2020 [35]	1/5(20)	3/5(60)	2/5(40)	-	2/5(40)	-	-	-
Hantoushzadeh, et al. 2020 [36]	7/7(100)	-	7/7(100)	-	4/9(44)	4/9(44)	-	-
Hirshberg, et al. 2020 [37]	5/5(100)	-	4/5(80)	-	4/5(80)	-	-	-
Qiancheng, et al. 2020 [38]	5/28(18)	-	7(25)	1 (3.6)	2 (7.1)	-	-	-
Hu, et al. 2020 [39]	4/7 (57.14)	-	2/7(29)	-	-	-	-	1/7(14)
Cooke, et al. 2020 [40]	1/2(50)	-	1/2(50)	-	1/2(50)	-	-	1/2(50)
Doria, et al. 2020 [41]	-	-	-	-	-	-	-	-
Lokken, et al. 2020 [42]	23/46(50)	-	32/46(70)	13/46(28)	20/46(44)	14/46(30)	12/46(28)	3/46(7)
Savasi, et al. 2020 [43]	41/77(53)	-	50/77(65)	-	19/77(25)	-	-	-
London, et al. 2020 [44]	26/68(38)	-	43/68(63)	-	-	-	-	-
Govind, et al. 2020 [45]	4/9(44)	-	8/9(89)	6/9(67)	4/9(44)	5/9(56)	-	-
Ferrazi, et al. 2020 [46]	20/42(48)	6/42(14)	18/42(43)	-	8/42(19)	-	-	2/42(5)

**Table 5:** Laboratory characteristics

Authors	Lymphocytopenia	Elavated CRP Concentration (mg/dl)	Confirmed SARS-CoV-2
Na Li et al. 2020 [11]			
Confirmed Case (16)	2/16 (12.5)	5/16 (31.3)	16/276 (6.0)
Suspected Case (18)	5/18(27.8)	11/18 (61.1)	
Control 2020(121)	15/121 (12.4)	68/121 (56.2)	
Control 2019(121)	14/121 (11.6)	57/121 (47.1)	
H.Chen, et al. 2020 [12]	5/9(56)	6/8(75)	9/9(100)
S.Chen. et al. 2020 [13]	4/5(80)	4/4(100)	5/5(100)
Zhu et al. 2020 [14]	-	-	9/9(100)
Yu, et al. 2020 [15]	5/7(71)	7/7(100)	7/7(100)
R.Chen, et al. 2020 [16]	25/41(61)	27/41(66)	16/41(39)
H.Liu, et al. 2020 [17]	25/41(61)	27/41(66)	16/41(39)
Yang, et al. 2020 [18]			
Confirmed (13)	-	-	13/13(100)
Control (42)	-	-	-
D.Liu, et al. 2020 [19]	12/15(80)	10/15(67)	15/15(100)
Yan, et al. 2020 [20]	51/116 (44.0)	51/116 (44.0)	65/116(56.0)
Breslin, et al. 2020a [21]	-	-	43/43(100)
Breslin, et al. 2020b [22]	-	-	7/7(100)
C.Wu, et al. 2020 [23]	5/8(62.5)	8/8(100)	6/6(100)
X.Wu, et al. 2020 [24]	-	-	19/23 (82.6)
Pierce-Wiliams, et al. 2020 [25]			
Severe (44)	-	43.3±45.9	64/64(100)
Critical (20)	-	141.9±88.6	
Khan, et al. 2020a [26]	-	2/3 (66.6)	3/3(100)
Fan, et al. 2020 [27]	2/2(100)	-	2/2(100)
Y.Chen, et al. 2020 [28]	4/4(100)	4/4(100)	4/4(100)
Campbell, et al. 2020 [29]	-	-	30/30(100)
Baergen, et al. 2020 [30]	-	-	20/20(100)
Khan, et al. 2020b [31]	4/17(24)	-	17/17(100)
Zheng, et al. 2020 [32]	-	-	33/33(100)
W.Liu, et al. 2020 [33]	-	-	19/19(100)
Cao, et al. 2020 [34]	1/10(10)	-	10/10(100)
Xu, et al. 2020 [35]	-	-	5/5(100)
Hantoushzadeh, et al. 2020 [36]	-	7/9(78)	7/9(78)
Hirshberg, et al. 2020 [37]	-	1/5(20)	5/5(100)
Qiancheng, et al. 2020 [38]	8 (28.6)	17(68)	28/28(100)
Hu, et al. 2020 [39]	-	-	7/7(100)
Cooke, et al. 2020 [40]	-	-	2/2(100)
Doria, et al. 2020 [41]	-	-	12/12(100)
Lokken, et al. 2020 [42]	-	-	46/46(100)
Savasi, et al. 2020 [43]	19/77(25)	39/77(51)	77/77(100)
London, et al. 2020 [44]	24/68 (35.3)	-	68/68(100)
Govind, et al. 2020 [45]	2/9(22)	-	9/9(100)
Ferrazi, et al. 2020 [46]	6/42(14)	17/42(40)	42/42(100)

**Table 6:** CT Scan findings

Authors	Unilateral pneumonia	Bilateral pneumonia	Typical viral infection
Na Li et al., 2020 [11]			
Confirmed Case (16)	8/16 (50.0)	7/16 (43.8)	-
Suspected Case (18)	10/18 (55.6)	7/18 (38.9)	-
Control 2020(121)	-	-	-
Control 2019(121)	-	-	-
H.Chen, et al. 2020 [12]	-	-	8/9(89)
S.Chen. et Al., 2020 [13]	2/5(40)	3/5(60)	-
Zhu et al. 2020 [14]	-	-	9/9(100)
Yu, et al. 2020 [15]	6/7(86)	1/7(14)	-
R.Chen, et al. 2020 [16]	12/41(29)	26/41(63)	-
H.Liu, et al. 2020 [17]	12/41(29)	26/41(63)	-
D.Liu, et al. 2020 [19]	-	-	15/15(100)
Yan, et al. 2020 [20]	-	-	104/116 (96.3)
C.Wu, et al. 2020 [23]	-	-	6/8(75)
X.Wu, et al. 2020 [24]	-	-	23/23(100)
Khan, et al. 2020a [26]	-	3/3(100)	-
Fan, et al. 2020 [27]	2/2(100)	-	-
Y.Chen, et al. 2020 [28]	-	4/4(100)	-
Cao, et al. 2020 [34]	4/10(40)	6/10(60)	-

**Table 7:** Treatment

Authors	Treatment				
	Steroid	Antibiotic	Antiviral	High throughput O2	ECMO
Na Li et al. 2020 [11]					
Confirmed Case (16)	-	16/16 (100.0)	4/16 (25.0)	-	-
Suspected Case (18)	-	18/18 (100.0)	-	-	-
Control 2020(121)	-	-	-	-	-
Control 2019(121)	-	-	-	-	-
H.Chen, et al. 2020 [12]	-	9/9(100)	6/9(67)	9/9(100)	-
Yu, et al. 2020 [15]	-	7/7(100)	7/7(100)	7/7(100)	-
D.Liu, et al. 2020 [19]	-	15/15(100)	11/15(73)	14/15(93)	-
Yan, et al. 2020 [20]	37/116 (31.9)	109/116 (94.0)	63/116 (54.3)	6/116 (5.2)	1/116 (0.9)
C.Wu, et al. 2020 [23]	-	8/8(100)	-	-	-
Pierce-Wiliams, et al. 2020 [25]					
Severe (44)	4/44 (9%)	22/44 (50%)	3/44 (7%)	5/44 (11%)	-
Critical (20)	11/20 (55%)	14 /20 (70%)	13/20 (65%)	11/20 (55%)	-
Khan, et al. 2020a [26]	-	3/3(100)	3/3(100)	-	-
Fan, et al. 2020 [27]	2/2(100)	2/2(100)	2/2(100)	-	-
Khan, et al. 2020b [31]	-	17/17(100)	16/17(94)	-	-
Xu, et al. 2020 [35]	4/5(80)	5/5(100)	5/5(100)	-	-
Hantoushzadeh, et al. 2020 [36]	-	9/9(100)	9/9(100)	-	-
Hirshberg, et al. 2020 [37]	5/5(100)	-	5/5(100)	-	-
Qiancheng, et al. 2020 [38]	-	24/28 (85.7)	20/28 (71.4)	-	-
Hu, et al. 2020 [39]	-	-	1/7(14)	-	-
Savasi, et al. 2020 [43]	-	27/77(35)	25/77(32)	20/77(26)	-

**Table 8:** Maternal comorbidities and maternal outcome

Authors	Chronic Illness	Pregnancy Complications	Maternal ICU Admission	Maternal Mortality
Na Li et al. 2020 [11]				
Confirmed Case (16)	2/16 (12.5)	11/16 (68.8)	-	-
Suspected Case (18)	1/18 (5.6)	13/18 (72.2)		
Control 2020(121)	5/121 (4.1)	38/121 (31.4)		
Control 2019(121)	-	32/121 (33.3)		
H.Chen, et al. 2020 [12]	-	7/9(77)	-	-
S.Chen. et al. 2020 [13]	-	3/5(60)	-	-
Zhu et al. 2020 [14]	-	4/9(44)	-	-
Yu, et al. 2020 [15]	2/7(29)	3/7(43)	-	-
R.Chen, et al. 2020 [16]	-	8/17(47)	-	-
H.Liu, et al. 2020 [17]	-	7/41(17)	-	-
Yan, et al. 2020 [20]	-	18/116(15.5)	8/116(6.9)	-
Breslin, et al. 2020a [21]	-	-	2/29 (4.7)	-
Pierce-Williams, et al. 2020 [25]				
Severe (44)	20/44(45)	9/44(20)	-	-
Critical (20)	7/20(35)	3/20(15)	-	-
Khan, et al. 2020b [31]	-	5/17 (29.4)	-	-
Zheng, et al. 2020 [32]	-	3/33(9)	-	-
Cao, et al. 2020 [34]	-	3/10(30)	-	-
Hantoushzadeh, et al. 2020 [36]	3/9(33)	-	7/9(77)	7/9(77)
Hirshberg, et al. 2020 [37]	5/5(100)	-	-	-
Qiancheng, et al. 2020 [38]	6/28 (21.4)	4/28 (14.3)	-	-
Cooke, et al. 2020 [40]	2/2(100)	-	2/2(100)	-
Lokken, et al. 2020 [42]	17/46(37)	-	8/46(17)	-
Savasi, et al. 2020 [43]	-	-	14/77(18)	-
London, et al. 2020 [44]	-	4/68(6)	-	-
Ferrazi, et al. 2020 [46]	-	6/42(14)	4/42(10)	-

**Table 9:** Neonatal outcomes

Authors	Intrauterine Fetal Death	Intrauterine Fetal Distress	Mean Birth Weight	Low Birth Weight	APGAR score at 1 minute after birth	APGAR score at 5 minutes after birth
Na Li et al. 2020 [11]						
Confirmed Case (16)	-	2/16 (12.5)	3066 .7 ± 560 .2	3/16 (17.6)	9.6 ± 0.5	10.0 ± 0.0
Suspected Case (18)		1/18 (6.0)	3198 .7 ± 522.6	2/18 (11.0)	9.6 ± 0.5	10.0 ± 0.0
Control 2020(121)		6 /121(5.0)	3317.1 ± 455.3	3/121 (2.5)	9.8 ± 0.4	10.0 ± 0.0
Control 2019(121)		6/121 (5.0)	3307.9 ± 419.3	3/121 (2.5)	9.9 ± 0.3	10.0 ± 0.0
H.Chen, et al. 2020 [12]	-	-	3011 grams	2/9(22)	9	10
S.Chen. et al. 2020 [13]	-	-	3691 grams	-	10	10
Zhu et al. 2020 [14]	-	6/9(67)	2423 grams	6/9(67)	9	9.4
Yu, et al. 2020 [15]		-	3264 grams	-	9	10
R.Chen, et al. 2020 [16]	-	-	3030 grams	-	9	10
Yang, et al. 2020 [18]						
Confirmed (13)	-	-	3063.2 ± 536.4	-	-	-
Control (42)	-	-	3317.1 ± 522.5	-	-	-
D.Liu, et al. 2020 [19]	-	-	-	-	8	9
Yan, et al. 2020 [20]	-	9 /99(10.6)	3108 ± 526	-	9	10
Pierce-Williams, et al. 2020 [25]						
Severe (44)	-	-	2945.8±509.2	-	-	8.8±0.8
Critical (20)	-	-	1924.6±846.6	-	-	7.2±2.0
Khan, et al. 2020a [26]	-	-	3373	-	8.6	9.6
Fan, et al. 2020 [27]	-	-	3145	-	9	10
Y.Chen, et al. 2020 [28]	-	-	3400	-	8	8
Campbell, et al. 2020 [29]	-	-	3370	-	-	-
Baergen,et al. 2020 [30]	-	-	3309	3/17(18)	8	9
Khan, et al. 2020b [31]	-	-	3104	-	9	9
Zheng, et al. 2020 [32]	-	-	-	-	-	-
W.Liu, et al. 2020 [33]	-	-	3293	-	8	9
Cao, et al. 2020 [34]	-	-	3003	2/10(20)	9	10
Xu, et al. 2020 [35]	-	-	2992	1/5(20)	8	9
Hantoushzadeh, et al. 2020 [36]	3/11(27)	3/11(27)	2464	5/11(45)	8	9
Hirshberg, et al. 2020 [37]	-	-	1818	3/3(100)	6	7
Qiancheng, et al. 2020 [38]	-	1/23(4)	-	1/23(4)	10	10
Hu, et al. 2020 [39]		-	3331	-	8	9
Cooke, et al. 2020 [40]	-	-	1450	2/2(100)	4	6
Doria, et al. 2020 [41]	-	-	2960	3/10(30)	9	10
Savasi, et al. 2020 [43]		-	3160	-	-	10
London, et al. 2020 [44]	-	1/55(2)	-	-	-	-
Govind, et al. 2020 [45]		-	3155	1/9(11)	7	9
Ferrazi, et al. 2020 [46]		-	3018	-	-	9
